# Prospective deployment of AI-based risk stratification to enable expedited mammography workflow in a safety-net setting

**DOI:** 10.1038/s41746-026-02743-x

**Published:** 2026-05-18

**Authors:** Maggie Chung, Eric Davis, Heather Greenwood, Jessica Hayward, Shinn-Huey Shirley Chou, Bonnie Joe, Loretta Strachowski, Tatiana Kelil, Rita Freimanis, Elissa Price, Kimberly Ray, Amie Lee, Adam Yala

**Affiliations:** 1 Department of Radiology and Biomedical Imaging, University of California, San Francisco, CA USA; 2 Computational Precision Health, University of California, Berkeley and University of California, San Francisco, Berkeley, CA USA

**Keywords:** Cancer, Diseases, Health care, Medical research, Oncology

## Abstract

Delays between screening and diagnostic mammography contribute to disparities in breast cancer outcomes and reduce screening adherence. We prospectively evaluated the operational feasibility and impact of integrating AI risk stratification into screening workflows at an urban safety net facility. In this HIPAA-compliant, IRB-approved, controlled study, Mirai 1-year risk scores were generated in real time from screening mammograms. Patients in the top 10% of risk were flagged as high-risk and, on enrollment days, were offered immediate screening interpretation and same-day diagnostic evaluation when indicated. Outcomes included operational feasibility, care delivery timelines for screening results, diagnostic evaluation, and biopsy, and cancer detection rate, compared with high-risk controls on non-enrollment days. Among 4145 screening mammograms, 525 were flagged as high-risk and 100 women consented to expedited care, of whom 94 percent received immediate reads and 26 received same-day diagnostic evaluation. Time to screening results, diagnostic evaluation, and biopsy were significantly shorter in expedited patients versus control patients with screen-detected cancers, with reductions of 99.1%, 99.1%, and 87.2%, respectively. The expedited cohort demonstrated a cancer detection rate of 60/1000 compared with 2.3/1000 among non-high-risk participants. Integrating AI risk models into clinical workflows substantially improved care delivery timelines and provides a translational framework to improve care and reduce disparities.

## Introduction

Timely follow-up after an abnormal screening mammogram is critical for early breast cancer detection. Screening mammograms are typically interpreted offline in batches after the patient has left the facility. If an abnormality is identified, the patient is contacted to return on a separate day for diagnostic imaging, and, if warranted, scheduled for biopsy at a later time. This multistep process introduces cumulative delays that increase patient anxiety, lower satisfaction, and contribute to delays in cancer care^1–3^. Women facing transportation challenges, inflexible work schedules, or caregiving responsibilities are disproportionately affected^4^. Black, Hispanic, and Asian women, in particular, experience longer delays to diagnostic evaluation and treatment, which exacerbates disparities in breast cancer outcomes^5–11^.

Prior work has demonstrated that same-day evaluation of screening abnormalities improves patient experience and adherence to future screening recommendations^12–14^. Expedited workflows have also been associated with reduced disparities in breast cancer care^15–17^. However, workflow constraints such as staffing limitations, mammography unit/room availability, and unpredictable daily volumes can make immediate screening interpretation and same-day diagnostic evaluation for all patients impractical or unfeasible in most screening programs. In addition, such an approach may not be cost-effective when applied universally^18,19^.

Risk-stratified triage for expedited “on-line” screening and diagnostic mammogram interpretation offers a potential solution. Traditional questionnaire-based risk models such as Gail and Tyrer–Cuzick typically provide 10-year or lifetime risk estimates, but do not calculate short-interval (i.e., 1-year) risk, which is most relevant for expedited interpretation^20^. In addition, they are time-consuming to collect, depend on patient recall, and are susceptible to language barriers. Artificial intelligence (AI) risk models that assess risk directly from screening mammograms can output risk scores in real-time without additional cumbersome steps. By directing limited resources toward women with high 1-year breast cancer risk scores, programs may maintain operational feasibility while offering expedited assessment for those who are most likely to benefit.

To this end, we conducted a prospective study at an urban safety-net hospital to evaluate the operational feasibility and care-delivery impact of offering expedited screening mammogram assessment to women at the top 10th percentile of 1-year breast cancer risk based on an AI risk model. We utilized Mirai, a widely validated mammogram-based AI risk model^21,22^. We hypothesized that this strategy would substantially shorten time to screening mammogram result and diagnostic evaluation while preserving workflow capacity by limiting expedited evaluations to a manageable subset of high-risk patients. This study was conducted in a large urban safety-net healthcare system serving a diverse population with a high proportion of publicly insured and uninsured patients. In this setting, timely diagnostic follow-up is often limited by patient scheduling and access barriers. These system-level constraints are common in safety-net environments and can lead to delays between screening and diagnostic evaluation despite prompt image interpretation. Within this context, risk-stratified triage for expedited assessment may offer a mechanism to more effectively align limited resources with patients most likely to benefit.

## Results

During the study period, 4145 screening mammograms were performed in 4145 women. Of these, 973 (23.5%) were performed on enrollment days and 3172 (76.5%) were performed on control days. Demographics were similar between enrollment and control days (Table 1).

**Table 1:** Demographics of enrollment versus non-enrollment days

	All		Enrollment days		Non-enrollment days		p
*n*	4145		973		3172		
Female (*n*)	4145		973		3172		
Mean Age (SD)	56.2 yrs (9.8 yrs)		56.3 yrs (9.6 yrs)		56.2 yrs (9.3 yrs)		0.74
Age Group (years)	n	%	n	%	n	%	
30–39	21	0.51%	4	0.41%	17	0.54%	0.90
40–49	1159	27.96%	261	26.82%	898	28.31%	
50–59	1294	31.22%	316	32.48%	978	30.83%	
60–69	1273	30.71%	302	31.04%	971	30.61%	
70–79	390	9.41%	88	9.04%	302	9.52%	
≥80	8	0.19%	2	0.21%	6	0.19%	
Breast Density	*n*	%	*n*	%	*n*	%	*p*
A	185	4.46%	55	5.65%	132	4.16%	0.20
B	1568	37.83%	355	36.49%	1212	38.21%	
C	2061	49.72%	481	49.43%	1578	49.75%	
D	331	7.99%	82	8.43%	250	7.88%	

Using the pre-calibrated threshold, the Mirai model flagged 525 (12.7%) of all 4145 patients as high-risk (i.e., top 10th percentile of 1-year risk). Of the 973 women on enrollment days, 115 (11.8%) were flagged as high-risk. Of the 3172 women on control days, 410 (12.9%) were flagged as high-risk. There was no significant difference in proportion of women flagged as high-risk on enrollment versus non-enrollment days (*p* = 0.41).

Of the 115 patients flagged as high-risk on enrollment days, 2 were not eligible due to participation in another clinical trial. Five could not be reached in time by the research coordinator while they were consenting or coordinating care for another patient. Eight patients did not consent to receive an immediate assessment. In total, 100 participants (mean 59.1 years ± 9.5 years) consented to expedited assessment. Of note, one participant was consented the morning after her screening mammogram owing to a one-time technical problem that delayed transmission of the high-risk notification to the research coordinator until the following day, which was also an enrollment day. She was contacted when the high-risk notification was received, provided informed consent, and returned for same-day diagnostic assessment as her screening mammogram was assessed as BI-RADS 0.

### Time to screening result, diagnosis, and biopsy

Median time from screening mammogram to screening result (Ts) was significantly shorter for all enrolled expedited participants (13.0 min vs. 191.9 min; *p* < 0.001) and for enrolled expedited participants with screen-detected cancers (12.23 min vs. 655.86 min; *p* < 0.001) compared to high-risk controls on non-enrollment days (Tables 2 and 3). Median time from screening mammogram to completion of diagnostic evaluation (Td) was significantly shorter for all enrolled expedited participants (1.27 h vs. 852.78 h; *p* < 0.001) and for expedited participants with screen-detected cancers (1.25 vs. 1535.85 h; *p* = 0.003) compared to high-risk controls. Median time from screening mammogram to completion of biopsy was significantly shorter for all expedited participants (20.11 days vs. 58.96 days; *p* < 0.001) and for expedited participants with screen-detected cancers (5.61 vs. 73.60 days).

**Table 2:** Workup times for expedited high-risk patients vs. high-risk controls

		Expedited high-risk patients	High-risk controls	p-value
Screening Mammogram to Screening Result (minutes)	*n*	94	410	
	Median	13.0	191.9	<0.001
	IQR	9.2, 19.8	84.1, 1207.8	
	Mean	14.7	900.9 (15.0 h)	<0.001
	95% CI	13.2, 16.1	769.8, 1030.8	
Screening Mammogram to Completion of Diagnostic Evaluation (hours)	n	30	79	
	Median	1.27	852.8 (35 days 12.78 h)	<0.001
	IQR	0.9, 2.2	498.3, 1661.0	
	Mean	16.0	1078.4 (44 days 22.4 h)	<0.001
	95% CI	−4.3, 36.3	872.9, 1283.9	
Screening Mammogram to Completion of Biopsy (days)	*n*	13	37	
	Median	20.1	59.0	<0.001
	IQR	5.2, 28.1	38.0, 96.7	
	Mean	18.4	69.9	<0.001
	95% CI	9.2, 27.6	54.8, 85.0

**Table 3:** Workup times for screen-detected cancers

		Expedited high-risk patients	High-risk controls	p-value
	*n*	6	10	
Screening Mammogram to Screening Result (minutes)	Median	12.2	655.9 (10.9 h)	<0.001
	IQR	11.4, 16.1	79.7, 2708.3	
	Mean	13.5	1461.6 (24.4 h)	0.03
	95% CI	9.7, 17.2	170.9, 2752.3	
Screening Mammogram to Completion of Diagnostic Evaluation (hours)	Median	1.3	1535.9 (64.0 days)	0.003
	IQR	1.0, 2.0	527.7, 1735.8	
	Mean	12.3	1375.1 (57.3 days)	0.003
	95% CI	−16.0, 40.5	615.1, 2135.0	
Screening Mammogram to Completion of Biopsy (days)	Median	5.6	73.6	0.003
	IQR	1.2, 16.9	52.8, 100.2	
	Mean	9.5	73.8	<0.001
	95% CI	−1.64, 20.6	45.6, 102.0	

**Fig. 1:**
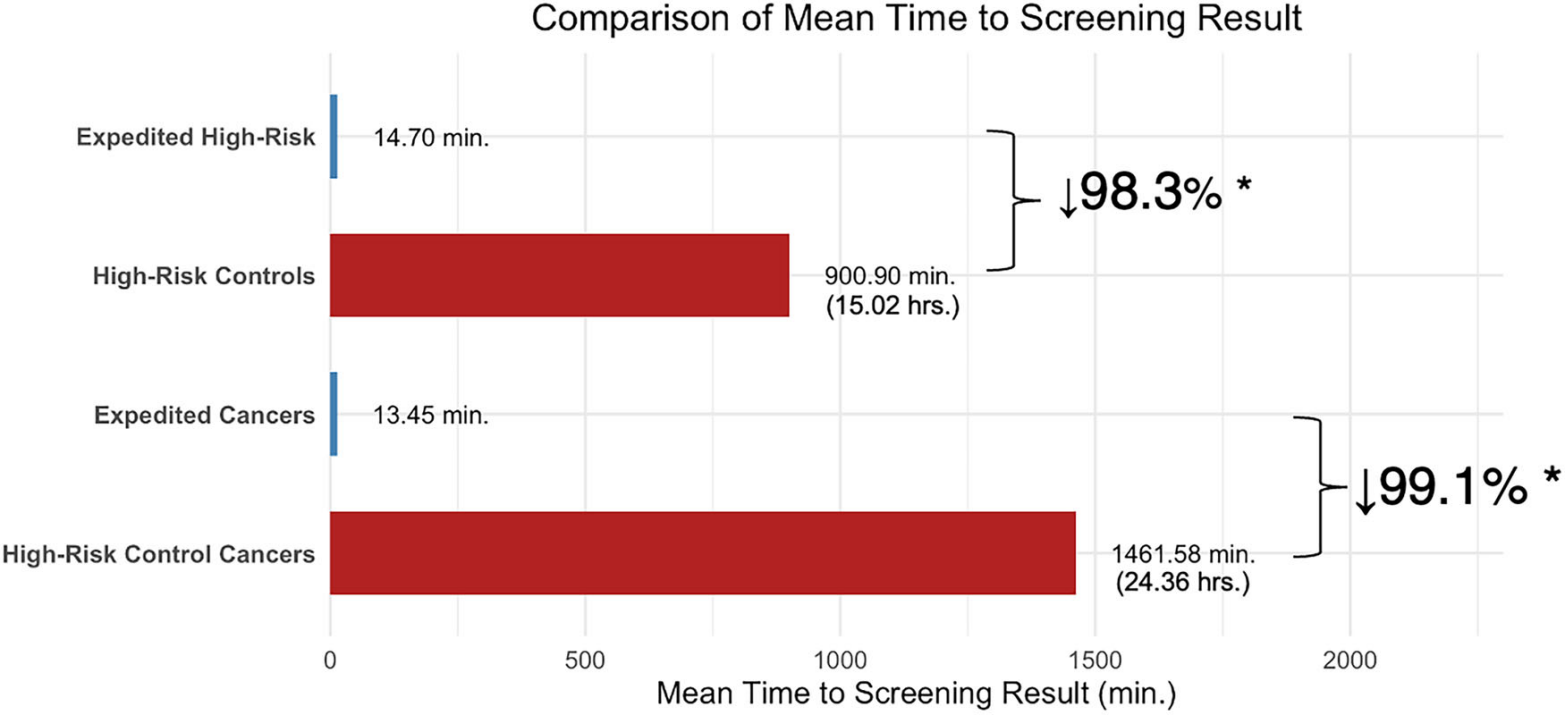
Comparison of mean time to screening result (Ts). AI enabled expedited assessment dramatically reduced the mean time to screening result for both high risk patients and cancers compared with high risk control groups, with reductions of 98.3 percent and 99.1 percent respectively.

**Fig. 2:**
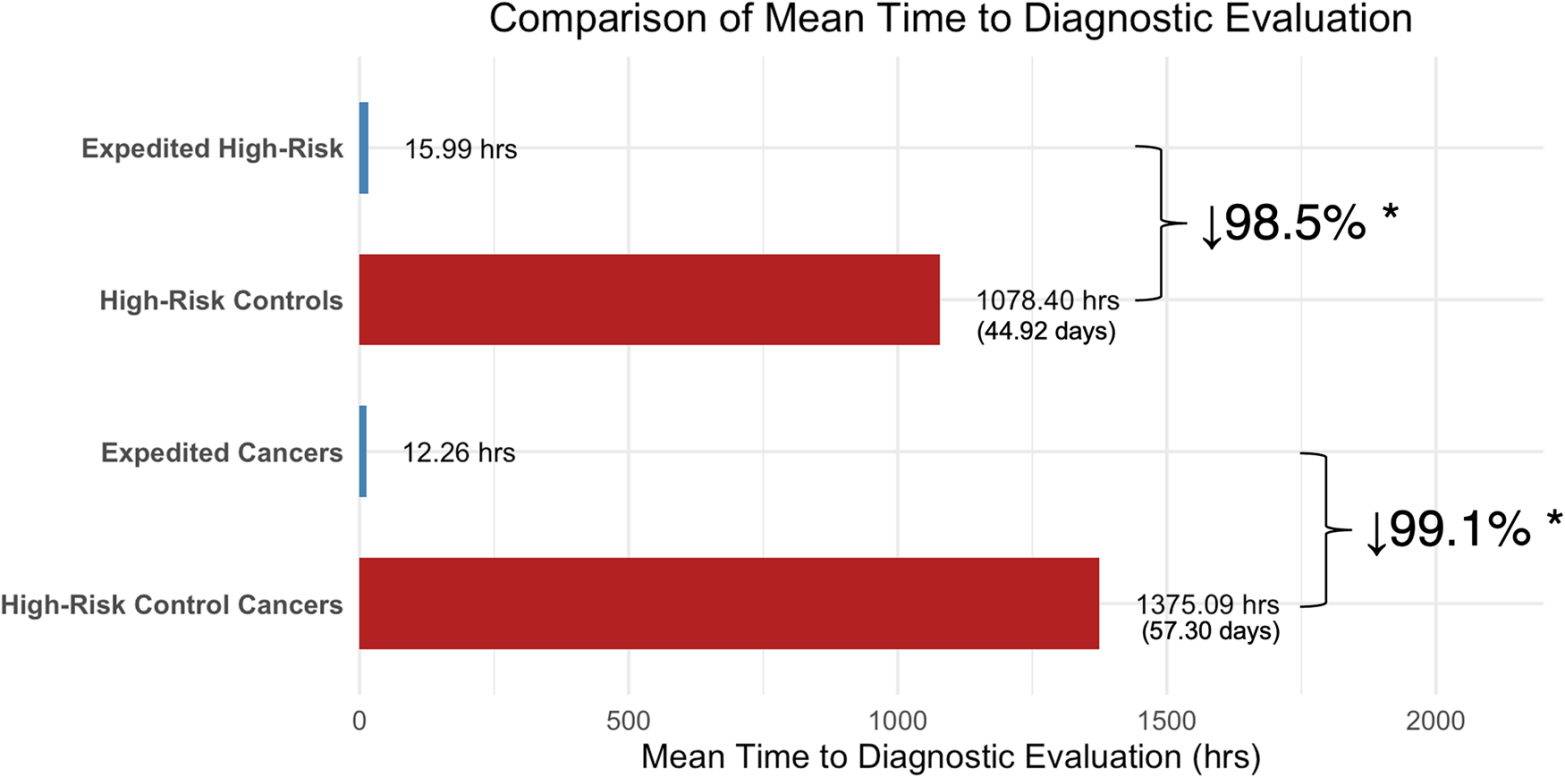
Comparison of mean time to diagnostic evaluation (Td). AI enabled expedited assessment substantially reduced the time from screening to diagnostic evaluation for both high risk patients and cancers compared with high risk controls, with reductions of 98.5 percent and 99.1 percent respectively.

**Fig. 3:**
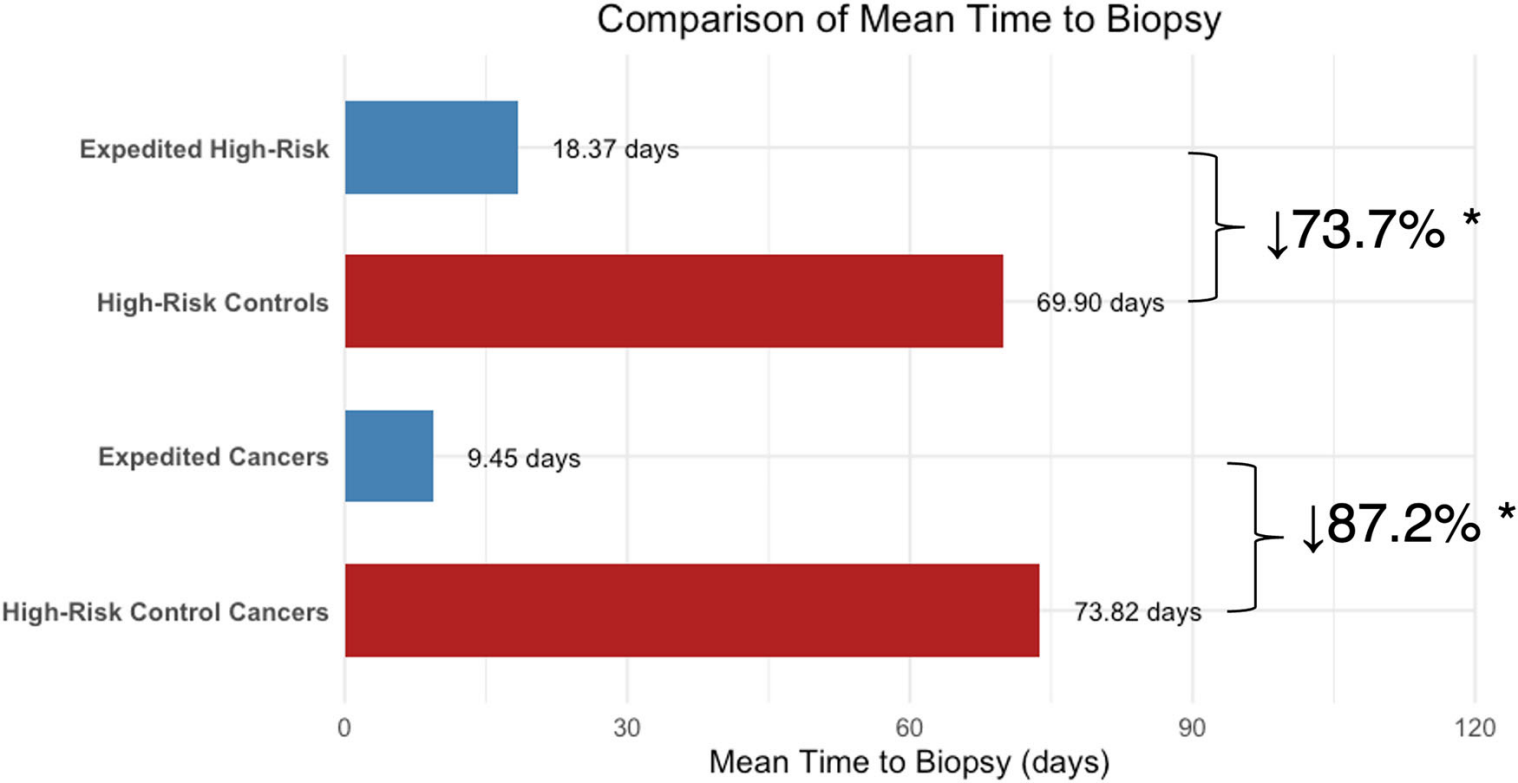
Comparison of mean time to biopsy (Tb). AI enabled expedited assessment substantially reduced the time from screening to biopsy for both high risk patients and cancers compared with high risk controls, with reductions of 73.7 percent and 87.2 percent respectively.

Similarly, mean times were significantly shorter for expedited participants compared to high-risk controls on non-enrollment days (Tables 2 and 3) (Figs. 1, 2, and 3). For all expedited participants, mean Ts was reduced by 98.3% (14.7 vs 900.9 min; *p* < 0.001), Td by 98.5% (16.0 vs 1078.4 h; *p* < 0.001), and Tb by 73.7% (18.4 vs 69.9 days; *p* < 0.001). Among expedited participants with screen-detected cancers, reductions were even greater: Ts was reduced by 99.1% (13.5 vs 1461.6 min; *p* = 0.03), Td by 99.1% (12.3 vs 1375.1 h; *p* = 0.003), and Tb by 87.2% (9.5 vs 73.8 days; *p* < 0.001).

Expediting high-risk patients on enrollment days did not significantly affect work-up times for non-expedited patients. For screening time (Ts), median values were 166.47 min (IQR 66.45–999.31) for non-expedited, non-high-risk patients on enrollment days and 152.60 min (IQR 66.63–1064.63) on control days (*p* = 0.50); corresponding mean values were 871.49 min and 858.63 min (*p* = 0.88). For diagnostic time (Td), median values were 889.33 h (IQR 383.76–1773.14) on enrollment days and 955.66 h (IQR 498.51–1752.90) on control days (*p* = 0.60), with mean values of 1274.59 h and 1322.33 h, respectively (*p* = 0.82). For biopsy time (Tb), median values were 45.80 days (IQR 33.93–67.02) on enrollment days and 48.98 days (IQR 25.79–70.89) on control days (*p* = 0.78), with mean values of 52.06 days and 54.96 days, respectively (*p* = 0.77).

### Workflow feasibility

Of the 100 enrolled participants, 94 received immediate screening interpretation; six did not receive immediate reads due to clinical time constraints (*n* = 4) and patient wait time (*n* = 2). Among participants who received immediate screening interpretation, 97.9% (92/94) received their screening result within 30 min. Of the 31 enrolled participants who were assigned BI-RADS 0 assessment at immediate screening interpretation, 26 (83.9%) underwent diagnostic imaging on the same day. Five (16.1%) required scheduling on a different day due to workflow limitations. Of these, 4 received their diagnostic evaluation 1, 2, 3, and 12 days later, respectively. One did not return for diagnostic evaluation. Two who underwent same-day diagnostic imaging also underwent same-day biopsy at the discretion of the interpreting radiologist.

### Outcomes in high-risk enrolled vs. high-risk control patients

Of the 6 participants who did not receive immediate screening interpretation due to workflow constraints, they had their screening exams interpreted by standard workflow and all 6 were assessed as BI-RADS 1 or 2. Of the 94 participants who received immediate screening assessments, 31 were assessed as BI-RADS 0 and 30 completed diagnostic evaluation. At diagnostic evaluation, 14 were assessed as BI-RADS 1 or 2, 3 were assessed as BI-RADS 3, 12 as BI-RADS 4, and 1 as BI-RADS 5. All 13 women assessed as BI-RADS 4 or 5 underwent biopsy, yielding 6 malignant, 2 high-risk, and 5 benign pathologies (Supplementary Fig. 1). Of the 6 malignant pathologies, 4 were invasive carcinomas (Supplementary Figs. 2–5) and 2 were ductal carcinomas in situ (Supplementary Figs. 6–10).

The CDR for this high-risk cohort was 60.0/1000 (6/100) (95% CI 22.3, 126.0), which was significantly higher compared to CDR of 2.3/1000 (2/858)(95% CI 0.28, 8.4)(*p* < 0.001) in the non-high-risk group on enrollment days, consistent with risk-based enrichment. Odds ratio was 27.1 (95% CI 4.8, 276.8). PPV1, PPV2, and PPV3 for the high-risk enrolled participants were 19.4% (6/31), 46.2% (6/13), and 46.2% (6/13), respectively (Table 4). Using an intention-to-treat approach, immediate interpretation of every 16.7 AI-flagged screening mammograms resulted in expedited care for a participant with a subsequent screen-detected cancer (6 cancers among 100 flagged high-risk participants). Likewise, offering expedited diagnostic evaluation to every 5.2 recalled high-risk participants resulted in one additional cancer being diagnosed more promptly (6 cancers among 31 recalled, high-risk participants).

**Table 4:** Screening and diagnostic outcomes in consented high-risk vs. high-risk controls

	All	Expedited high-risk	High-risk controls	*p*
*n*	4145	100	410	
Recall Rate	12.8% (532/4145)	31.0% (31/100)	21.5% (88/410)	0.04*
CDR	6.0/1000 (25/4145)	60.0/1000 (6/100)	24.4/1000 (10/410)	0.10
% Diagnostic Exams Performed	89.1% (474/532)	96.8% (30/31)	89.8% (79/88)	0.45
Diagnostic Assessment				
BI-RADS 1 or 2	47.7% (226/474)	46.7% (14/30)	31.7% (25/79)	0.14
BI-RADS 3	22.4% (106/474)	10.0% (3/30)	19.0% (15/79)	0.26
BI-RADS 4	29.5% (140/474)	40.0% (12/30)	48.1% (38/79)	0.45
BI-RADS 5	0.4% (2/474)	3.3% (1/30)	1.3% (1/79)	0.47
PPV1	4.7% (25/532)	19.4% (6/31)	11.4% (10/88)	0.26
PPV2	17.6% (25/142)	46.2% (6/13)	25.6% (10/39)	0.17
PPV3	18.3% (25/137)	46.2% (6/13)	27.0% (10/37)	0.20

Of the 410 high-risk control patients on non-enrollment days, 21.5% were recalled (88/410), which was significantly lower compared to that in high-risk enrolled participants (31/100; *p* = 0.04). Of those, 79 underwent diagnostic evaluation. Fifteen were assessed as BI-RADS 3, 38 as BI-RADS 4, and 1 as BI-RADS 5. The CDR for this cohort was 24.4/1000 (10/410; 95% CI 11.8, 44.4), which did not differ significantly from that of the high-risk enrolled group (*p* = 0.10). PPV1, PPV2, and PPV3 for high-risk controls were 11.4% (10/88), 25.6% (10/39), and 27.0% (10/37), respectively (Table 4).

Across both enrollment and non-enrollment days, the Mirai model flagged an enriched subset that included 68.0% (17/25) of screen-detected cancers. Of the 15 patients who were flagged as high-risk on enrollment days but who were not enrolled, one resulted in cancer (invasive ductal carcinoma). On enrollment days, Mirai flagged 77.8% (7/9) of screen-detected cancers. On non-enrollment days, it flagged 62.5% (10/16) of screen-detected cancers.

## Discussion

In this prospective study, integration of an AI-based risk model for triaging enabled immediate interpretation and expedited diagnostic evaluation for women with high 1-year risk scores. The most pronounced benefit was observed among expedited participants with screen-detected cancers, for whom time to screening result, diagnostic result, and biopsy were reduced by 99.1%, 99.1%, and 87.2%, respectively, compared to control patients on non-enrollment days. Such reductions have the potential to accelerate treatment and improve clinical outcomes^23^. For high-risk patients with negative exams, expedited interpretation can shorten the period of uncertainty, improve the screening experience, and potentially improve adherence to annual screening recommendations in high-risk women^13^.

Although short-interval cancer detection models and short-term risk models can exhibit substantial overlap in predictive performance for 1-year cancer outcomes, we intentionally selected a risk-based approach for operational reasons. Risk model outputs can be translated into patient-level percentiles across the screening population, which allows thresholds to be selected to tailor expedited workflows to match anticipated capacity. In addition, we used a risk model solely for prioritization and did not provide image-level decision support to radiologists in order to minimize automation and anchoring bias. As such, the observed cancer detection metrics should be interpreted as a reflection of the enriched population rather than as interpreted as improved diagnostic performance.

Our results highlight the importance of thoughtful, workflow-oriented design to enable feasible implementation. Threshold selection is central to the workflow impact of risk-stratification strategies: flagging too large a proportion of patients risks overwhelming diagnostic capacity, whereas flagging too few may limit benefit. In our study, applying a 10th-percentile Mirai threshold for 1-year risk enriched the expedited cohort while flagging 12.7% of patients for expedited evaluation. Nearly all flagged participants (94%) were able to receive immediate screening reads and most (83.8%) completed same-day diagnostic evaluation, supporting the operational feasibility of this approach. Importantly, the specific percentile threshold used here was selected to align with local diagnostic capacity and workflow constraints. Implementation in other settings should involve threshold calibration based on local screening volume, staffing, and downstream diagnostic capacity. Maintaining a relatively small expedited cohort also minimizes disruption to standard workflows for non-expedited patients. Consistent with this, we observed no significant differences in work-up times for non-expedited, non-high-risk patients between enrollment and non-enrollment days.

These findings build on prior work by Friedewald et al., which used an AI model to triage mammograms for expedited evaluation^24^. While they retrospectively calibrated their threshold to identify 14% of patients as high-risk, the threshold flagged 29% of patients in prospective implementation. We prospectively applied the 10th-percentile threshold determined from retrospective validation, and the observed rate of 12.7% aligned closely with the anticipated proportion. This more specific threshold promotes workflow feasibility. In our study, 83.9% (26/31) of participants with BI-RADS 0 findings at immediate screening mammography received same-day diagnostic evaluation, compared with 72.7% (24/33) reported by Friedewald et al. In our study, immediate interpretation of every 16.7 AI-flagged mammograms (6 cancers among 100 flagged) resulted in expedited care for one participant with screen-detected cancer, compared with 43.7 mammograms (3/131) in their study. Similarly, offering expedited diagnostic evaluation to every 5.2 recalled high-risk participants (6/31) yielded one additional promptly diagnosed cancer, versus 11.0 (3/33) in their study. This supports that our 10th percentile threshold using the Mirai model is a more efficient strategy to prioritize patients with cancer.

Notably, recall rates among high-risk enrolled patients in our study was high (31.0%). This likely reflects the higher proportion of cancers in this patient subset as well as the prospective design. Radiologists may have been more inclined to recommend additional imaging when they are aware of the participant’s elevated risk status and when the participant was already present, eliminating the need for a return visit. Importantly, despite this high recall rate, PPV1–3 remained high (19.4%, 46.2%, and 46.2%, respectively) compared with that in the overall population (4.7%, 17.6%, and 18.3%) and high-risk controls (11.4%, 25.6%, and 27.0%), supporting that the elevated recall rate reflected appropriate identification of cancers in high-risk patients rather than unnecessary workup. Together, these findings underscore that modified workflows should be compared with current standard workflows to assess the impact on downstream metrics.

By prospectively validating AI-based triaging for screening programs, this study provides evidence for how risk-based tools can be translated into clinical workflows. Our findings are particularly relevant at a time when many commercial AI applications are increasingly marketed directly to patients on a pay-for-access basis, raising concerns that such approaches may exacerbate socioeconomic inequities in breast cancer care. In contrast, we demonstrate the integration of Mirai, a free and open-source AI model, within existing clinical workflows. By lowering barriers to patient access, this approach may help reduce disparities and narrow gaps in breast cancer outcomes. The clinical implications of these findings may be greatest in safety-net settings, where vulnerable populations often face barriers to timely follow-up and are at higher risk of being lost to care. By helping patients who are most likely to have cancer receive expedited evaluation, AI-based triage may mitigate disparities in access to and timeliness of care and improve outcomes^15,17,23^. This study did not include a sufficiently large non-disadvantaged comparison group. As such, it was not designed or powered to assess whether the intervention reduced disparities between demographic subgroups. Rather, the intervention targets delayed diagnostic evaluation following abnormal screening, a well-established contributor to disparities in breast cancer outcomes. By reducing time to diagnostic resolution for patients most likely to have cancer within a high-risk population, the intervention addresses a pathway through which delayed management contributes to adverse outcomes.

Our study has several limitations. It was conducted at a single safety-net institution, potentially limiting generalizability. Because the intervention was designed around local workflow constraints and staffing models, threshold selection and feasibility may differ across institutions and would require local calibration. Patients were followed for at least three months to capture diagnostic and biopsy outcomes; cancers diagnosed beyond this interval were not included. Enrollment days were pre-specified based on research coordinator availability rather than random assignment. However, enrollment days spanned multiple weekdays and were not selected based on scheduled screening volume or patient characteristics. Demographics were similar between enrollment and non-enrollment control days. Workflow feasibility is likely to vary across sites and will depend on each breast screening program’s volumes and operational capacity. We evaluated a single high-risk threshold; future studies will be needed to systematically evaluate how different risk thresholds and workflow designs influence recall burden, false positives, and downstream utilization across diverse practice settings. Although analyses of interval and subsequent screen-detected cancers would further support cancer enrichment in the high-risk population, this study was not powered for comparison of these outcomes; larger studies with longer follow-up are needed. Future studies incorporating additional demographic information (e.g., ethnicity, socioeconomic status) and access-related data across diverse practice settings will be needed to directly assess how socioeconomic and access factors influence enrollment and downstream outcomes. Finally, patients with a history of mastectomy, current pregnancy, or breast implants were not enrolled. While quantitative measures of radiologist and patient experience were beyond the scope of this study, radiologists reported that the expedited workflow integrated smoothly into routine practice without requiring additional staffing. They valued the flexibility to defer expedited assessment when it was not feasible to avoid workflow disruption. From the patient perspective, many participants expressed appreciation for receiving results sooner, which helped alleviate anxiety and, for some, expedited their cancer diagnosis. Patients’ preference for expedited assessment was reflected in the high enrollment rate (87%) observed in the study.

In summary, this prospective study demonstrates that an AI-based risk model can be feasibly integrated into screening workflows to enable immediate interpretation and same-day diagnostic evaluation for high-risk women. This approach substantially reduced delays to diagnosis and biopsy, with potential to improve outcomes, lessen anxiety, and support adherence to follow-up. By prospectively evaluating operational feasibility and care-delivery timelines, our findings provide a framework for translating AI risk-stratification tools into screening programs.

## Methods

### Ethics statement

This HIPAA-compliant study protocol was approved by the institutional review boards of University of California, San Francisco (IRB number 23-40218). All procedures conformed to the Declaration of Helsinki and relevant regulations. Written informed consent was obtained from all participants.

### Study design

We conducted a prospective, controlled study at an urban safety-net hospital from December 2024 through June 2025. Per the clinicaltrials.gov checklist, this study is exempt from National Clinical Trial (NCT) registration, as it does not satisfy all four required criteria for classification as a clinical trial^25^.

### Study setting

The study was conducted at a large urban safety-net hospital that serves a predominantly socioeconomically disadvantaged population. Ninety-two percent of patients receive publicly funded health insurance (Medicare or Medicaid) or are uninsured. The patient population is racially and ethnically diverse, with individuals identifying as Latine/x (43%), Asian (20%), White (16%), and Black (13%). More than 30% of patients report a primary language other than English, most commonly Spanish or Cantonese^26^.

As a baseline reference, during the pre-intervention period from January 1, 2024 to June 30, 2024, the screening program performed a monthly average of 646 screening examinations, or approximately 31 screening mammograms per workday. The screening program routinely adds on 0–2 same day diagnostic evaluation based on clinical need or patient request.

### AI risk algorithm

In the prospective study, the Mirai breast cancer risk model was used to generate 1-year risk scores immediately after screening mammograms were obtained. The algorithm processes digital standard craniocaudal and mediolateral oblique views from bilateral 2D screening mammography and outputs non-normalized risk scores. Risk scores were obtained from all patients during the study period. On enrollment days, risk scores were used solely to select patients for enrollment and were not used to guide clinical interpretation or downstream management. On non-enrollment days, radiologists and patients were not notified of flagged studies. A retrospective analysis of 114,229 local screening mammograms (2006–2023) was performed to calibrate the Mirai risk threshold based on anticipated workflow feasibility, identifying the top 10th percentile as high-risk (estimated CDR ~ 60/1000); full methodology, including a priori estimates of sample size and power, is provided in the Supplementary Note 1.

### Risk stratification and recruitment

Eligible participants were women aged 30 years or older who underwent screening mammography and were flagged by the Mirai algorithm as high-risk (top 10th percentile of 1-year risk). Patients were excluded if they had prior history of mastectomy, implants, or were pregnant. On 1–2 designated enrollment days per week, eligible high-risk patients were approached for enrollment. Informed consent was obtained from participants prior to expedited assessments.

### Expedited and standard imaging workflows

**Fig. 4:**
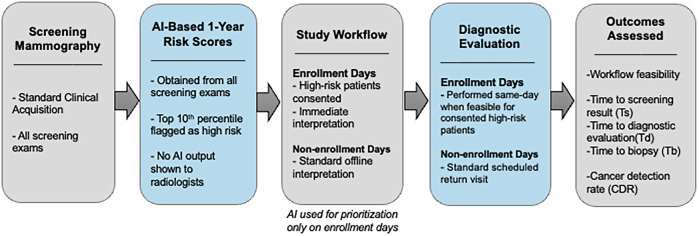
AI-enabled workflow for expedited mammography assessment. This diagram illustrates how AI derived one year risk scores from screening exams identify high risk patients for same day interpretation and diagnostic workup on enrollment days, compared with standard scheduling on non enrollment days, and summarizes the primary workflow and clinical outcomes assessed including feasibility, time to results, and cancer detection.

On enrollment (intervention) days, consented high-risk women received immediate screening mammogram interpretation (Fig. 4). Interpreting radiologists were informed that women were prioritized based on Mirai risk, but were not provided the risk scores nor shown any AI localization or heatmaps. At immediate screening mammogram interpretation, patients who received BI-RADS 0 assessments were offered same-day, expedited diagnostic evaluation if feasible based on clinical workflow. If not feasible, diagnostic imaging was scheduled based on next clinical availability. Biopsies were scheduled and performed per standard of care. If warranted, same-day biopsy could be performed at the discretion of the interpreting radiologist. Enrollment days were pre-specified based on research coordinator availability and varied across days of the week.

On non-enrollment (control) days, screening examinations followed the standard of care workflow and were read offline after patients left the facility. Patients with abnormal screening mammograms were contacted directly by the screening program to schedule a return appointment for diagnostic imaging. If the diagnostic assessment is suspicious (BI-RADS 4 or 5), a biopsy is scheduled prior to the patient leaving the diagnostic visit, although the biopsy itself is typically performed on a subsequent date.

On both enrollment and non-enrollment days, standard mammographic views (craniocaudal and mediolateral oblique) were obtained (Selenia Dimensions, Hologic). Images were interpreted by one of 11 board-certified, fellowship-trained breast radiologists with 2–35 years of experience. The BI-RADS assessments at screening mammography and, if performed, diagnostic evaluation were recorded.

### Outcomes and data collection, statistical analysis

To assess the clinical benefit of AI-based triaging for immediate interpretation, we measured three time intervals: (1) from screening mammogram to screening result (Ts), (2) from screening mammogram to diagnostic evaluation (Td), and (3) from screening mammogram to biopsy (Tb). For expedited participants, the time of screening result release was defined as when results were verbally communicated by the study coordinator to the participant; in the standard workflow for non-enrolled patients, result release was the time the radiologist signed the screening report. For all patients, Td and Tb were defined as the completion times of diagnostic imaging and biopsy examinations, respectively.

Patients were followed for at least three months from the date of their screening mammogram to assess diagnostic and biopsy outcomes. Screening outcomes assessed were recall rate, PPV1 (defined as cancers per number of recalled exams), PPV2 (defined as cancers per number of diagnostic exams with a biopsy recommended), PPV3 (defined as cancers per number of biopsies performed), and cancer detection rate (CDR).

To evaluate the clinical feasibility of the AI-based expedited workflow, we recorded how many enrolled participants received immediate interpretation of their screening mammogram. Among patients who received a BI-RADS 0 assessment, we recorded whether diagnostic imaging was completed on the same day. If immediate screening reads or, when recommended, same-day diagnostic evaluation could not be performed, the reason was recorded: clinical workflow constraints (e.g., radiologist or technologist unavailability) or patient-related factor (e.g., patient unavailability).

Medians with interquartile ranges were reported as the primary summary statistics given the skewed distributions of time intervals. Means with 95% confidence intervals were estimated. Group comparisons of Ts, Td, and Tb were performed with the Mann–Whitney U test, and differences in means were evaluated using Welch’s two-sample *t* test. Group comparisons for recall rate, CDR, and PPV1–3 were performed using chi-square tests; Fisher’s exact test was used in comparisons with small counts. A two-sided *P* < 0.05 was considered statistically significant. Statistical analysis was performed by M.C. using R version 4.5.1^27^.

## Supplementary information

Supplementary Information

## Data Availability

The dataset generated and/or analyzed during the current study are not publicly available due to patient privacy protections and institutional restrictions on sharing identifiable clinical imaging data, but are available from the corresponding author on reasonable request.

## References

[1] Loving, V. A., Aminololama-Shakeri, S. & Leung, J. W. T. Anxiety and its association with screening mammography. *J. Breast Imaging* **3**, 266–272 (2021). 38424779 10.1093/jbi/wbab024

[2] Brett, J., Bankhead, C., Henderson, B., Watson, E. & Austoker, J. The psychological impact of mammographic screening. A systematic review. *Psychooncology* **14**, 917–938 (2005). 15786514 10.1002/pon.904

[3] Brocken, P., Prins, J. B., Dekhuijzen, P. N. R. & van der Heijden, H. F. M. The faster the better?—A systematic review on distress in the diagnostic phase of suspected cancer, and the influence of rapid diagnostic pathways. *Psychooncology* **21**, 1–10 (2012). 22905349 10.1002/pon.1929

[4] Lawson, M. B. et al. Multilevel factors associated with time to biopsy after abnormal screening mammography results by race and ethnicity. *JAMA Oncol.* **8**, 1115–1126 (2022). 35737381 10.1001/jamaoncol.2022.1990 PMC9227677

[5] Miller-Kleinhenz, J. M. et al. Racial disparities in diagnostic delay among women with breast cancer. *J. Am. Coll. Radiol.* **18**, 1384–1393 (2021). 34280379 10.1016/j.jacr.2021.06.019 PMC8492512

[6] Daly, B. & Olopade, O. I. A perfect storm: how tumor biology, genomics, and health care delivery patterns collide to create a racial survival disparity in breast cancer and proposed interventions for change. *Ca. Cancer J. Clin.* **65**, 221–238 (2015). 25960198 10.3322/caac.21271

[7] Press, R., Carrasquillo, O., Sciacca, R. R. & Giardina, E.-G. V. Racial/ethnic disparities in time to follow-up after an abnormal mammogram. *J. Women’s. Health* **17**, 923–930 (2008). 10.1089/jwh.2007.0402 PMC2942754 18554094

[8] Selove, R. et al. Time from screening mammography to biopsy and from biopsy to breast cancer treatment among black and white, women medicare beneficiaries not participating in a health maintenance organization. *Women’s. Health Issues* **26**, 642–647 (2016). 27773529 10.1016/j.whi.2016.09.003 PMC5116399

[9] Sheinfeld Gorin, S. N. & Heck, J. E. Delay in breast cancer diagnosis by race/ethnicity. *J. Clin. Oncol.* **23**, 6004–6004 (2005).

[10] Molina, Y., Silva, A. & Rauscher, G. H. Racial/ethnic disparities in time to a breast cancer diagnosis: the mediating effects of health care facility factors. *Med. Care* **53**, 872–878 (2015). 26366519 10.1097/MLR.0000000000000417 PMC4570266

[11] Nguyen, K. H., Pasick, R. J., Stewart, S. L., Kerlikowske, K. & Karliner, L. S. Disparities in abnormal mammogram follow-up time for Asian women compared with non-Hispanic white women and between Asian ethnic groups. *Cancer* **123**, 3468–3475 (2017). 28603859 10.1002/cncr.30756 PMC5648644

[12] Barton, M. B. et al. Decreasing women’s anxieties after abnormal mammograms: a controlled trial. *J. Natl. Cancer Inst.* **96**, 529–538 (2004). 15069115 10.1093/jnci/djh083

[13] Dolan, N. C., McGrae McDermott, M., Morrow, M., Venta, L. & Martin, G. J. Impact of same-day screening mammography availability: results of a controlled clinical trial. *Arch. Intern. Med.* **159**, 393–398 (1999). 10030314 10.1001/archinte.159.4.393

[14] Shah, B. A., Mirchandani, A. & Abrol, S. Impact of same day screening mammogram results on women’s satisfaction and overall breast cancer screening experience: a quality improvement survey analysis. *BMC Women’s. Health* **22**, 338 (2022). 35941606 10.1186/s12905-022-01919-3 PMC9361536

[15] Dontchos, B. N. et al. Disparities in same-day diagnostic imaging in breast cancer screening: impact of an immediate-read screening mammography program implemented during the COVID-19 pandemic. *Am. J. Roentgenol.* **218**, 270–278 (2022). 34494449 10.2214/AJR.21.26597

[16] Oluyemi, E. Editorial comment: offering immediate screening mammography interpretation may be an effective way to reduce the racial and ethnic disparity gap in the time to diagnostic follow-up. *Am. J. Roentgenol.* **218**, 278 (2022). 34523956 10.2214/AJR.21.26798

[17] Dontchos, B. N. et al. Impact of a same-day breast biopsy program on disparities in time to biopsy. *J. Am. Coll. Radiol.* **16**, 1554–1560 (2019). 31152690 10.1016/j.jacr.2019.05.011

[18] Raza, S. et al. Patient expectations and costs of immediate reporting of screening mammography. *Am. J. Roentgenol.* **177**, 579–583 (2001). 11517050 10.2214/ajr.177.3.1770579

[19] Stewart, K. A., Neumann, P. J., Fletcher, S. W. & Barton, M. B. The effect of immediate reading of screening mammograms on medical care utilization and costs after false-positive mammograms. *Health Serv. Res.* **42**, 1464–1482 (2007). 17610433 10.1111/j.1475-6773.2006.00660.x PMC1955276

[20] Kim, G. & Bahl, M. Assessing risk of breast cancer: a review of risk prediction models. *J. Breast Imaging* **3**, 144–155 (2021). 33778488 10.1093/jbi/wbab001 PMC7980704

[21] Yala, A. et al. Toward robust mammography-based models for breast cancer risk. *Sci. Transl. Med.* **13**, eaba4373 (2021). 33504648 10.1126/scitranslmed.aba4373

[22] Yala, A. et al. Multi-institutional validation of a mammography-based breast cancer risk model. *J. Clin. Oncol.* **40**, 1732–1740 (2022). 34767469 10.1200/JCO.21.01337 PMC9148689

[23] Hanna, T. P. et al. Mortality due to cancer treatment delay: systematic review and meta-analysis. *BMJ* **371**, m4087 (2020). 33148535 10.1136/bmj.m4087 PMC7610021

[24] Friedewald, S. M. et al. Triaging mammography with artificial intelligence: an implementation study. *Breast Cancer Res. Treat.* **211**, 1–10 (2025). 39881074 10.1007/s10549-025-07616-7 PMC11953103

[25] NIH’s Definition of a Clinical Trial | Grants & Funding. https://grants.nih.gov/policy-and-compliance/policy-topics/clinical-trials/definition.

[26] Annual Reports. *Zuckerberg San Francisco General Hospital and Trauma Center* https://www.zuckerbergsanfranciscogeneral.org/services/annual-reports/.

[27] R: The R Project for Statistical Computing. https://www.r-project.org/.

