# Supplementary material for: Prospective deployment of AI-based risk stratification to enable expedited mammography workflow in a safety-net setting
Source: NPJ Digit Med. 2026 May 18;9:603. doi: 10.1038/s41746-026-02743-x (PMC13448717; doi:10.1038/s41746-026-02743-x)
Supplement: Supplementary file 1 [file 41746_2026_2743_MOESM1_ESM.docx]

Supplementary Materials

Supplementary note 1.
Retrospective Calibration and Sample Size Calculation
To identify a risk threshold for high-risk triage and to inform sample size estimation, we retrospectively obtained Mirai breast cancer risk assessments from 114,229 bilateral 2D digital screening mammograms acquired between January 2006 and January 2023 from our local institution. Exams were considered positive if there was histopathologic diagnosis of breast cancer within 12 months. Exams were considered negative if there was at least 12 months of follow-up without histopathologic diagnosis of breast cancer. Based on this retrospective cohort, patients in the top 10th percentile of Mirai risk scores had an estimated cancer detection rate (CDR) of approximately 60/1000.

Based on an a priori power calculation using cancer detection rates of 60 per 1,000 in the high-risk group and 6 per 1,000 in the standard group, a total sample size of 100 patients for the prospective enrollment was estimated to provide 90% power to detect a significant difference at a two-sided alpha of 0.05.

Supplementary Figure 1. Cancer Outcomes and Mirai Risk Status on Enrollment and Non-enrollment days

Flow diagram showing cancer outcomes among 4,145 screening patients stratified by Mirai AI risk status and study enrollment.

Supplementary Figure 2. 48-year-old woman presenting for screening mammogram.
Left craniocaudal and mediolateral oblique views from screening mammogram flagged as high-risk by Mirai. Immediate screening mammogram assessment identified architectural distortion with associated calcifications in the left lower inner quadrant (circle).

Supplementary Figure 3. 48-year-old woman presenting for screening mammogram. Same day diagnostic mammogram and ultrasound were performed. Diagnostic left breast ultrasound demonstrated architectural distortion spanning 7:00, 4-6 cm from the nipple. Within the architectural distortion, there are two discrete similar appearing masses, the dominant (arrows) measuring up to 1.4 cm, assessed as BI-RADS 4.

Supplementary Figure 4. 48-year-old woman presenting for screening mammogram. Based on level of suspicion and staffing availability, same day ultrasound guided core biopsy was performed. Pathology yielded invasive ductal carcinoma.

Supplementary Figure 5. 48-year-old woman presenting for screening mammogram. Contrast enhanced breast MRI performed 9 days later demonstrated a 2.6 cm irregularly shaped mass (circle) with spiculated margins and heterogeneous internal enhancement in the left breast, lower inner quadrant. Kinetics curve assessment demonstrated a fast initial and delayed persistent enhancement pattern.

Supplementary Figure 6. 40-year-old woman presenting for baseline screening mammogram. Craniocaudal mammogram, with corresponding digitally zoomed-in views, demonstrates calcifications (circled) in the right outer central breast.

Supplementary Figure 7. 40-year-old woman presenting for baseline screening mammogram. Mediolateral oblique mammogram, with corresponding digitally zoomed-in views, demonstrates calcifications (circled) in the right outer central breast.

Supplementary Figure 8. 40-year-old woman presenting for baseline screening mammogram. Same day diagnostic evaluation was performed. Spot magnification craniocaudal and lateral views demonstrate amorphous calcifications (circles) in the right breast, outer central quadrant, anterior to middle third, spanning 25 mm, assessed as BI-RADS 4.

Supplementary Figure 9. 40-year-old woman presenting for baseline screening mammogram. Stereotactic biopsy of the calcifications yielded ductal carcinoma in situ.

Supplementary Figure 10. 40-year-old woman presenting for baseline screening mammogram. Contrast enhanced breast MRI was performed. Subtraction images demonstrate 10.3 cm non-mass enhancement (circle) with linear distribution and clumped internal enhancement in the right breast, outer central breast. Kinetic curve assessment demonstrates fast initial and delayed washout enhancement pattern.

Consort Checklist

**CONSORT 2025 checklist of information to include when reporting a randomised trial***

| **Section / Topic** | **No** | **CONSORT 2025 checklist item description** | **Reported on page no.** |
| --- | --- | --- | --- |
| **Title and abstract** | | |  |
| Title and structured abstract | 1a | Identification as a randomised trial n | n/a |
|  | 1b | Structured summary of the trial design, methods, results, and conclusions | 2 |
| **Open science** | | |  |
| Trial registration | 2 | Name of trial registry, identifying number (with URL) and date of registration | n/a |
| Protocol and statistical analysis plan | 3 | Where the trial protocol and statistical analysis plan can be accessed | n/a |
| Data sharing | 4 | Where and how the individual de-identified participant data (including data dictionary), statistical code and any other materials can be accessed | 17 |
| Funding and conflicts of interest | 5a | Sources of funding and other support (e.g., supply of drugs), and role of funders in the design, conduct, analysis and reporting of the trial | 18 |
|  | 5b | Financial and other conflicts of interest of the manuscript authors | 18 |
| **Introduction** | | |  |
| Background and rationale | 6 | Scientific background and rationale | 3 |
| Objectives | 7 | Specific objectives related to benefits and harms | 4 |
| **Methods** | | |  |
| Patient and public involvement | 8 | Details of patient or public involvement in the design, conduct and reporting of the trial | n/a |
| Trial design | 9 | Description of trial design including type of trial (e.g., parallel group, crossover), allocation ratio, and framework (e.g., superiority, equivalence, non-inferiority, exploratory) | 13 |
| Changes to trial protocol | 10 | Important changes to the trial after it commenced including any outcomes or analyses that were not prespecified, with reason | n/a |
| Trial setting | 11 | Settings (e.g., community, hospital) and locations (e.g., countries, sites) where the trial was conducted | 14 |
| Eligibility criteria | 12a | Eligibility criteria for participants | 15 |
|  | 12b | If applicable, eligibility criteria for sites and for individuals delivering the interventions (e.g., surgeons, physiotherapists) | n/a |
| Intervention and comparator | 13 | Intervention and comparator with sufficient details to allow replication. If relevant, where additional materials describing the intervention and comparator (e.g., intervention manual) can be accessed | 15 |
| Outcomes | 14 | Pre-specified primary and secondary outcomes, including the specific measurement variable (e.g., systolic blood pressure), analysis metric (e.g., change from baseline, final value, time to event), method of aggregation (e.g., median, proportion), and time point for each outcome | 16 |
| Harms | 15 | How harms were defined and assessed (e.g., systematically, non-systematically) | 17 |
| Sample size | 16a | How sample size was determined, including all assumptions supporting the sample size calculation | Supplementary note 1 |
|  | 16b | Explanation of any interim analyses and stopping guidelines | n/a |
| Randomisation: |  |  |  |
| Sequence generation | 17a | Who generated the random allocation sequence and the method used | n/a |
|  | 17b | Type of randomisation and details of any restriction (e.g., stratification, blocking and block size) | n/a |
| Allocation concealment mechanism | 18 | Mechanism used to implement the random allocation sequence (e.g., central computer/telephone; sequentially numbered, opaque, sealed containers), describing any steps to conceal the sequence until interventions were assigned | n/a |
| Implementation | 19 | Whether the personnel who enrolled and those who assigned participants to the interventions had access to the random allocation sequence | n/a |
| Blinding | 20a | Who was blinded after assignment to interventions (e.g., participants, care providers, outcome assessors, data analysts) | n/a |
|  | 20b | If blinded, how blinding was achieved and description of the similarity of interventions | n/a |
| Statistical methods | 21a | Statistical methods used to compare groups for primary and secondary outcomes, including harms | 16 |
|  | 21b | Definition of who is included in each analysis (e.g., all randomised participants), and in which group | 16 |
|  | 21c | How missing data were handled in the analysis | n/a |
|  | 21d | Methods for any additional analyses (e.g., subgroup and sensitivity analyses), distinguishing prespecified from post-hoc | 16 |
| **Results** | | |  |
| Participant flow, including flow diagram | 22a | For each group, the numbers of participants who were randomly assigned, received intended intervention, and were analysed for the primary outcome | 5 |
|  | 22b | For each group, losses and exclusions after randomisation, together with reasons | 5 |
| Recruitment | 23a | Dates defining the periods of recruitment and follow-up for outcomes of benefits and harms | 13 |
|  | 23b | If relevant, why the trial ended or was stopped | n/a |
| Intervention and comparator delivery | 24a | Intervention and comparator as they were actually administered (e.g., where appropriate, who delivered the intervention/comparator, how participants adhered, whether they were delivered as intended [fidelity]) | 5 |
|  | 24b | Concomitant care received during the trial for each group | 5 |
| Baseline data | 25 | A table showing baseline demographic and clinical characteristics for each group | 23 |
| Numbers analysed,  outcomes and estimation | 26 | For each primary and secondary outcome, by group:   - the number of participants included in the analysis - the number of participants with available data at the outcome time point - result for each group, and the estimated effect size and its precision (such as 95% confidence interval) - for binary outcomes, presentation of both absolute and relative effect size | 27 |
| Harms | 27 | All harms or unintended events in each group | 7 |
| Ancillary analyses | 28 | Any other analyses performed, including subgroup and sensitivity analyses, distinguishing pre-specified from post-hoc | 7 |
| **Discussion** | | |  |
| Interpretation | 29 | Interpretation consistent with results, balancing benefits and harms, and considering other relevant evidence | 9 |
| Limitations | 30 | Trial limitations, addressing sources of potential bias, imprecision, generalisability, and, if relevant, multiplicity of analyses | 10 |

*We strongly recommend reading this statement in conjunction with the CONSORT 2025 Explanation and Elaboration and/or the CONSORT 2025 Expanded Checklist for important clarifications on all the items. We also recommend reading relevant CONSORT extensions. See [www.consort-spirit.org](http://www.consort-spirit.org).

Citation: Hopewell S, Chan AW, Collins GS, Hróbjartsson A, Moher D, Schulz KF, et al. CONSORT 2025 Statement: updated guideline for reporting randomised trials. BMJ. 2025; 388:e081123. <https://dx.doi.org/10.1136/bmj-2024-081123>.

© 2025 Hopewell et al. This is an Open Access article distributed under the terms of the Creative Commons Attribution License (<https://creativecommons.org/licenses/by/4.0/>), which permits unrestricted use, distribution, and reproduction in any medium, provided the original work is properly cited.
